# Vlogger's persuasive strategy and consumers' purchase intention: The dual mediating role of para-social interactions and perceived value

**DOI:** 10.3389/fpsyg.2022.1080507

**Published:** 2022-12-07

**Authors:** Xiayan Sheng, Zhenhua Zeng, Wen Zhang, Yuanhui Hu

**Affiliations:** ^1^School of Literature and Arts, Jiangxi Normal University, Nanchang, China; ^2^School of Journalism and Communication, Jiangxi Normal University, Nanchang, China; ^3^School of Psychology, Beijing Normal University, Beijing, China

**Keywords:** vlogger, Aristotle's three appeals of persuasion, para-social interactions, perceived value, purchase intention

## Abstract

It is a new advertising marketing method for commodity companies to use vloggers to endorse their products, and to influence consumers' attitudes and decisions. In view of this phenomenon, there are few studies on the relationship between vloggers and consumers, and this study aims to explore how the persuasive strategies used by vloggers to promote products influence consumers' purchase intentions. Based on the concepts of Aristotle's persuasion theory, this study extracts two specific persuasive approaches, “two-sided messages” and “emotional appeal,” to explore consumers' perceptions of them and the effectiveness of these two strategies. At the same time, para-social interaction and perceived value as intermediary factors are also included in the study for further discussion. The study empirically analyzed a sample of 511 questionnaires from participants who had purchased products recommended by vloggers and came to the following conclusions: (1) vloggers can enhance consumers' purchase intention by adopting two-sided messages persuasion when promoting products; (2) vloggers' emotional persuasion can enhance consumers' purchase intention; (3) as an intermediary variable, para-social interaction plays a more obvious role in vloggers' persuasion by appealing to emotions. The audience can have common feelings with vloggers, and they are more connected with each other, thus increasing their willingness to buy; (4) Perceived value, as an intermediary variable, plays a more obvious role in vloggers' persuasion with the two-sided messages. The two-sided messages can show vloggers' credibility and more abundant information about products, so that consumers have a positive perception of product value, and the direct persuasion effect of the two-sided messages is greater. Based on the results of the study, this paper helps vloggers to adopt different persuasive approaches for different types of audiences, choose the proper marketing methods, attract the potential customers, and achieve such purposes as enriching product marketing forms and increasing market share.

## Introduction

Persuasion is an effective way to change attitudes. When persuasion is the overriding goal, the way of presentation may be more important than the content of its proposition (Mcquarrie and Mick, 1996). Carl Hovland, an American psychologist and communicator, once put forward the theory of communication persuasion, and pointed out that the source, the content of information, the characteristics of the receiver and the background of information are the four major factors that affect persuasion (Demirdöge, 2010). Aristotle also once put forward the classical persuasion theory, arguing that persuaders can achieve the purpose of persuasion through three rhetorical methods: “ethos,” “logos,” and “pathos” (Fortenbaugh, 2016). In daily life, advertising is a persuasive tool in mass communication, which aims to impress the target audience or attract potential consumers, so as to create the desired changes in the target market and better sell products (Çam, 2019). A major theme of advertising and consumer research is how persuasive information can change people's thoughts, feelings and actions, especially in purchasing goods and services (McGuire, 2000). Traditional advertising methods are mainly newspapers, TV, posters and celebrity endorsements. Nowadays, with the development of the Internet, there are a large number of users on the online media platform who are famous for their self-performance and have established a huge social group of fans (Xu and Pratt, 2018; Ki and Kim, 2019; Lou and Yuan, 2019). These people are called online vloggers, social media influencers or online celebrities. Therefore, more and more enterprises or brand companies begin to use these online celebrities or vloggers to endorse their products, and enhance consumers' brand awareness and purchase decisions, which is also a new advertising marketing method (Lou and Yuan, 2019). These vloggers often have influence, perceived authenticity and multi-identity characteristics (De Veirman et al., 2017; Audrezet et al., 2020; Schouten et al., 2020). They usually share their created content online, show their lives or share product preferences, establish contact with followers and take online social activities as their career. Vloggers often attract followers around a specific field, such as beauty influencer, fashionista, fitness guru, etc. (Ki and Kim, 2019). They are usually in the intermediary position between commodity companies and consumers. They promote products through marketing forms such as brand placement, product evaluation and in-depth experience in videos, and push users who agree with their lifestyle to become consumers of products. Vloggers can often get product-related information from companies before the product is released (Onishi and Manchanda, 2012). Therefore, vloggers can experience products before consumers, and then convey their feelings to consumers, so that consumers can get product information and experience before purchasing, which will influence their judgment and choice of products. In particular, para-social interaction emphasizes a kind of unilateral intimacy between the audience and media figures, which creates an “illusion” of a face-to-face personal relationship between individuals and vloggers (Chen et al., 2021). Positive para-social interaction will make consumers more willing to accept the content published by vloggers on social media and increase their participation in the content published by vloggers (Labrecque, 2014), thus affecting the formation of their purchase intention.

**Figure 1:**
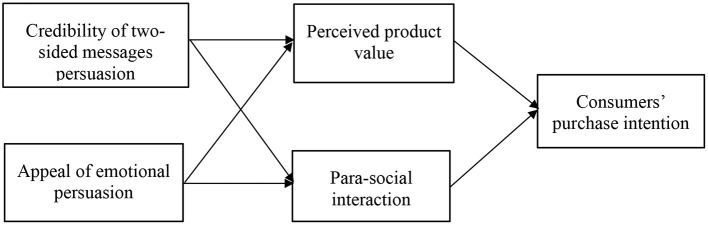
Theoretical model.

Aiming at the relationship between vloggers and consumers, Esber and Wong (2020) pointed out that the marketing method of vloggers promoting products to followers on social media has become a new field that traditional marketing managers pay attention to. Consumers in the new era are growing up in the booming environment of Internet and social media. They are more eager for fun, challenges and socializing, and tend to exchange information on social media (Faulds and Mangold, 2009). Therefore, it is worth exploring how to use the Internet to achieve positive product marketing and how product companies and vloggers can better attract and persuade consumers. Existing scholars have focused on how vloggers influence consumers' perceptions and purchase intentions of luxury brands (Lee and Watkins, 2016), consumers' trust in vloggers and their change of online shopping intention (Bayazit et al., 2017), the impact of Instagram influencers' number of fans on product marketing (De Veirman et al., 2017), and consumers' perception of vloggers' credibility (Chapple and Cownie, 2017; Hill et al., 2020). However, a review reveals that there is not yet a wealth of academic research on the relationship between vloggers and consumers, and little attention has been paid to the impact of vloggers' persuasion strategies on consumers. Therefore, this paper will draw on the classic Aristotle's persuasion theory to explore how vloggers' persuasive strategies affect consumers' purchase intention. At the same time, para-social interaction and perceived value often play an essential role in consumers' purchasing decisions (Dodds and Monroe, 1985; Labrecque, 2014), therefore, these two are included in this paper as mediating factors for further examination. This study attempts to explore the following questions: first, how can traditional Aristotle's three persuasion theories be applied to the persuasion strategies or methods of modern vloggers? Second, how do vloggers' persuasion strategies affect consumers' purchase intention? Thirdly, how do para-social interaction and perceived value play an intermediary role in the process of vloggers influencing consumers' purchase intention, and compare their intermediary role in different persuasion strategies. Figure 1 demonstrates the conceptual model of this study.

According to the above problems, this study begins with a theoretical analysis and literature review. Based on the concept of Aristotle's persuasion theory and the factors that affect vloggers' marketing, and extracts two specific persuasion strategies, namely “two-sided messages” and “emotional appeal,” to explore consumers' perception of them and the effectiveness of these strategies. Second, the study collected topic-related data through a questionnaire. Finally, quantitative analysis and modeling were used to test the hypothesis that the vlogger persuasion strategy affects consumers' purchase intentions, and conclusions were drawn. The possible contributions of this study include: Firstly, from the perspective of persuasion communication, it is beneficial to enrich the related research in the field of Internet marketing, reveal the internal relationship between language and consumer behavior, and hopefully provide helpful guidance for vloggers' marketing practice. Secondly, the intermediary of para-social interaction and perceived value helps to explain the transmission path that affects consumers' purchase intention and uncover the process of language effect. Finally, this study can help commodity companies and vloggers fully understand consumers' psychology, pay attention to the influence of different language styles on consumers' psychology, give weight to the use of appropriate language rhetoric in advertisements, attract potential customers, and guide consumers to enhance their purchase intention.

## Literature review and research hypothesis

### Literature review

#### Contemporary application of Aristotle's persuasion theory

Persuasion refers to the using specific strategies by individuals or groups to influence the perceptions and actions of others through the transmission of message, so that others accept their views and thus achieve the desired purpose (Amos et al., 2022). The act of persuasion has a wide scope, involving politics, culture, business, and daily interactions between people. Aristotle's theory of persuasion identifies three approaches, “ethos,” “logos,” and “pathos.” The process of persuading others is essentially the application of these three modes of persuasion (Amos et al., 2022).

First of all, “ethos” refers to the persuader's credibility. When the persuader is trustworthy and can win the audience's trust, the audience will accept their point of view and think that their ideas are authentic (Alkhirbash, 2016). Secondly, “logos” mainly refers to any rational appeal based on a logical conclusion. Persuaders try to convince the audience with a reasonable claim and provide appropriate evidence to support their statements (Murthy and Ghosal, 2014). Finally, “pathos” refers to the speaker's persuasion effect by mobilizing the audience's emotion, appealing to the audience's values and emotions and arousing the public's deep resonance.

Aristotle's theory of persuasion has been widely used in various research fields. Specifically, in the field of politics, Aristotle's three persuasion methods are often used to analyze the speeches of presidents and celebrities (Ghazani, 2016; Montgomery, 2017; Raissouni, 2020), as well as the effects of persuasion in political systems and texts (Brown et al., 2012). Persuasion is considered to be linguistically manipulative in that it is a strategy of unobtrusive discourse to subconsciously influence the listener's beliefs and attitudes. In addition, with the development of the Internet, Aristotle's persuasion theory has been applied to explore issues such as the effectiveness of persuasion in website design and e-commerce platforms (Chu et al., 2014; Tirdatov, 2014). In the field of advertising and consumers, McGuire (2000) analyzed figurative language, such as metaphors, similes, rhetorical questions, hyperboles, etc., and found that the message style variables of language have a certain influence on the perception and persuasion effect of communication. Lamichhane (2017) concluded by comparing Aristotle's three persuasion methods that the emotional appeal in advertising content and text information can influence advertising effect and consumer behavior more than the other two. Çam (2019) constructed Aristotle's persuasion framework, which summarized “ethos” as “expert, attractive, trustworthy, etc.,” “logos” as “narrative, testimony, comparison, etc.,” and “pathos” as “fear, excitement, reliance, etc.,” and analyzes the content of advertising rhetorical structure through the refinement of meaning. These are the innovative applications of traditional Aristotle's theory in contemporary research in various fields.

Thus, past research has confirmed the excellent applicability of Aristotle's persuasion theory. In the context of online influencer marketing, the expression or persuasion of vloggers in promoting products is an important means to connect and attract consumers and arouse their desire to buy. In the past, scholars generally studied Aristotle's persuasion theory in traditional television commercials or advertising texts, or focused on the rhetorical analysis of advertising language. This study turns its perspective to the discourse expressions of vloggers in social media, trying to provide new ideas and directions for the contemporary application of this theory.

#### Vlogger marketing and consumers

Vlogger's marketing relies on the rapid development of online media and the spread of information, which has become an efficient new marketing method and a new paradigm for information dissemination (Lou and Yuan, 2019; Stubb et al., 2019). In response to this phenomenon, existing scholars have studied the topic from three main perspectives:

The first is the study of credibility. Existing studies consider trust as one of the important factors affecting the effectiveness of vlogger marketing and analyze the construction of trust in vlogger marketing based on source credibility theory (De Veirman et al., 2017; Djafarova and Rushworth, 2017). Sparks and Browning (2011) stated that trust and consumer purchase decisions are positively related, with trust increasing consumers' perceived value of the merchant and serving to reduce perceived risk and promote purchase. The higher the level of trust the more likely consumers are to make a purchase. Perceived credibility plays a vital role in consumers' decision-making process and can reduce uncertainty. Recipients are more likely to be convinced by sources that they think are credible or attractive (McCroskey et al., 1974). Vloggers' generated content usually adds their personal characteristics and the information they post is usually not perceived as purely driven by commercial interests (Bao and Chang, 2014), which not only provides an enjoyable experience for followers, but also enhances the audience's trust in the product and their willingness to buy it (Breves et al., 2019). Research in this area has fully revealed the importance of trust in vlogger marketing.

The second is identity research. Identity is another important influencing factor of vlogger marketing put forward by researchers. When consumers find similarities between their image and that of the vlogger, such as shared interests, values and characteristics, it evokes similar feelings of identification, making them more likely to adopt the vlogger's beliefs, attitudes and behaviors (Schouten et al., 2020). This identification in turn has an impact on the subsequent behavior of the consumer. Accordingly, the generation of identity is more related to consumers' emotional activity and emotional perception.

The third is the study of para-social interaction. Para-social interaction originated from psychology, focusing on the relationship between Internet celebrities and consumers. Para-social interactions create an imaginary or real interaction between the consumer and the vlogger. Consumers can interact with vloggers and communicate their thoughts and feelings. Consumers give feedback on the content published by vloggers, including likes, comments, reposts, etc. (Dibble et al., 2016). Scholars have confirmed that the para-social interaction between vloggers and consumers will affect consumers' purchase intention (Jin and Ryu, 2020; Sokolova and Kefi, 2020), the para-social relationship between them will affect the establishment of perceived credibility (Reinikainen et al., 2020).

Based on the above, it can be seen that in past studies of the vlogger-consumer relationship, the audience's perception of vlogger's credibility, identification or cognitive emotions, and intimate interactions and relationships on the Internet all affect their acceptance of vlogger marketing. This study will also make new explorations and attempts from these perspectives, combining perceived values and correlating them for analysis.

### Research hypothesis

#### Persuasion strategies of “ethos” and “logos” and consumers' purchase intention

“Ethos” refers to the credibility and authority of the speaker, that is, the persuader makes the audience think that they have the right to speak on a certain issue by demonstrating their ability and experience (Powell et al., 2011). Vloggers usually have certain professional knowledge in specific fields, such as health care, tourism, food, fitness, make-up, fashion, etc. Djafarova and Rushworth (2017) pointed out that the professional skills and knowledge displayed by vloggers in specific fields will form the audience's trust in their recommended products. In addition, “logos” pays attention to the credibility of the persuasive content. The speakers usually demonstrate their views with the help of information, comparison, factual evidence and other elements, and weigh the pros and cons to the audience (Lamichhane, 2017). Thus, both “ethos” and “logos” emphasize credibility to a certain extent.

In advertising research, credibility is divided into “source credibility” and “content credibility.” This distinction was first put forward by communication scholar Rosenthal (1971), and then confirmed by Botan and Frey (1983). According to the message persuasion model, the credibility of the communicator or source is an important factor in its persuasive power, and people often judge the credibility of a message from the source and the content of the message (Hovland and Weiss, 1951). Factors such as the audience's cognitive ability and willingness will interact with the source and message content, and jointly affect the persuasion effect (Petty et al., 1983). A persuasive information source has many language strategies that can be used to explain information. The information source must make a choice whether to present one-sided messages (presenting only the product's benefits) or two-sided messages (presenting both the product's benefits and drawbacks) (Hale et al., 1991).

On the one hand, persuaders presenting two-sided messages can effectively enhance their credibility as information sources. For example, Hendriks et al. (2022) confirmed that when scientists provide two-sided messages in health communication, people's trust in the scientist will increase. Mayweg-Paus and Jucks (2018) also pointed out that experts can gather more consensus and gain more trust by taking a two-sided messages position to communicate. Winter and Krämer (2012) used ELM model to study that among the numerous and complicated information on the Internet, people would prefer those users who have professional knowledge and provide two-sided messages. Two-sided messages strategy contains tips on product defects or negative information. Consumers can easily think that advertisers are honest and their advertising claims are credible (Kamins and Assael, 1987).

On the other hand, two-sided messages provide more comprehensive factual evidence, which can enhance the credibility of the information content. Specifically, in the research on consumer behavior, many scholars think that two-sided messages is often more convincing (Golden and Alpert, 1987; Kamins et al., 1989; Sherman et al., 1991; Clemons et al., 2006). Ahluwalia et al. (2000) found that consumers usually think negative information is more valuable, and they will refer more to the influence of negative information in purchasing decisions. One-sided messages guarantee the quality of products because it is “too good to be true” and thus loses its effectiveness (Shimp and Bearden, 1982). When consumers convey richer, more positive and accurate data or information, they can make more informed choices and decisions (Hibbard and Peters, 2003; Berning et al., 2011; Wei and Miao, 2013).

Based on the above, two-sided messages, a type of information presentation, can cause audiences to perceive trust in both the communicator himself and the content of the communication and influence their consumption decisions. Therefore, this paper adopts the variable of two-sided messages as a persuasive factor and puts forward the following hypothesis:

*H1a: Consumers' perception of the credibility of vloggers' two-sided messages persuasion positively relates to their purchase intention*.

#### Persuasion strategy of “pathos” and consumers' purchase intention

“Pathos” is the persuasive power to stimulate or control the audience's psychological reaction. Emotion is an instinctive psychological state, which is usually triggered by some stimulating events, that is, the stimulation occurring in the organism will trigger psychological reactions such as liking, pleasure, impulsiveness and anxiety (Scherer, 2005). Emotional changes will lead to different judgments, and audiences will make different judgments under different emotions (Yi and Jai, 2020). Aristotle believed that speakers must be good at analyzing the psychology and emotions of different types of audiences in order to target their emotions and put the audience in a certain state of mind that the speakers expect, so that persuasion can be successful (Ghazani, 2016). Emotions are believed to play an important role in decision-making. Many of the choices people make are guided by emotions, while decisions and consequences can also produce various emotions (Brosch et al., 2013). In the study of consumer behavior, emotional factors are often valued by scholars. According to Gardner (1985), advertising content often affects consumers' emotional state by using emotional music, pictures or words. Consumers with more positive emotions are more likely to have impulsive buying behavior (Rook and Gardner, 1993). Among Aristotle's three persuasion strategies, “pathos” means that the persuader influences people's rational judgment by arousing the audience's emotion (Al-Momani, 2014), specifically appealing to fear, evoking sympathy, anger or joy, which are considered important persuasive tools to compensate for the lack of sensory experience of consumers and to create positive feelings about the product. De Pelsmacker and Geuens (1997) divided the emotional stimulation in advertisements into six categories: humor, warmth, nostalgia, eroticism, provocation and fear. He believed that advertisers used more elaborate “emotion-oriented” strategies to influence the audience's attitude toward products. Consumers' emotional state fluctuates before, during and after consumption, and emotions generally affect people's emotional decisions (Yi and Jai, 2020). Therefore, according to previous studies, vloggers' emotional persuasion can infect consumers, prompting them to have certain emotional reactions, and thus play a role in consumers' purchase intention and behavior. Based on this, this study makes the following hypothesis:

*H1b: The appeal of vloggers' emotional persuasion is positively related to consumers' purchase intention*.

#### The mediating effect of para-social interaction

Horton and Richard Wohl (1956) put forward the concept of para-social interaction (PSI). They think the new mass media gives people the illusion of face-to-face communication. The seemingly face-to-face relationship between the audience and performers is called para-social relationship, and para-social interaction is the degree to which media users regard media celebrities as close social partners. After the concept was put forward, scholars studied the para-social interaction between users and media figures in various backgrounds. For example, Levy (1979) studied the para-social relationship between TV news viewers and news figures, and pointed out that para-social interaction provided emotional satisfaction for viewers and encouraged them to continue watching programs. Hoffner (1996) explored the relationship between children and their favorite TV characters. Wenner (1976) confirmed that TV can accompany and compensate the face-to-face social interaction between the elderly and others, and help to reduce their loneliness. Auter and Palmgreen (2000) developed a new multi-dimensional social interaction scale based on previous research to test the relationship between viewers and TV roles. In recent years, PSI theory has been used to explore consumer behavior in the online environment, and mainly focuses on the relationship between digital celebrities and fans (Hills, 2015; Hsu, 2020), the influence of para-social interaction on brand evaluation (Zhang and Hung, 2020; Zhang et al., 2022), and how the para-social relationship between users and vloggers affects users' purchase intention (Kim et al., 2015).

As the main platform for consumers to communicate with online vloggers, social media breaks the time and space constraints, increases the frequency of interaction between consumers and vloggers, and provides the foundation for cultivating the para-social relationship between them. The relationship between consumers and vloggers further affects consumers' willingness to accept advertisements published by vloggers and their attitudes and behaviors toward products endorsed by vloggers. As consumers can communicate and interact directly with characters on social media and make comments like friends, this para-social interaction atmosphere helps to cultivate close social relationships between media celebrities and audiences (Labrecque, 2014; Chung and Cho, 2017), which in turn can reduce consumers' concerns and encourage them to purchase. That is to say, the media platform shortens the distance between ordinary users and vloggers, and provides a possibility for ordinary users to communicate with Internet celebrities and form social relationships. The closer this relationship is, the more likely consumers are to buy products recommended by vloggers. The existing research also proves that para-social interaction has become the mechanism of social media's influence on its users (Colliander and Dahlén, 2011; Yuan et al., 2016; Gong and Li, 2017). Therefore, para-social interaction is a particularly appropriate and useful perspective to explain the influence of vloggers on the audience from the perspective of consumers' emotions. According to the above analysis, the following hypotheses are put forward:

*H2a: Para-social interactions has a mediating effect between consumers' perception of the credibility of vloggers' two-sided messages persuasion and their purchase intention*.
*H2b: Para-social interactions has a mediating effect between the appeal of vloggers' emotional persuasion and consumers' purchase intention*.
*H2c: The mediating effect of para-social interaction between vloggers' emotional persuasion and consumers' purchase intention is greater than that between the credibility of two-sided messages persuasion and purchase intention*.

#### The mediating effect of perceived value

Dodds and Monroe (1985) studied the relationship among three structures: perceived quality, perceived value and purchase intention, and pointed out that perceived value affects consumers' purchase behavior, and they are more willing to buy products with high perceived value. When consumers buy products, they will subjectively perceive and judge the value of products (Zeithaml, 1988). In Internet marketing, consumers can get useful product information through the introduction of vloggers, such as the practicality, reliability and usability of products, and then form the value perception of target products through value judgment. In the process of value judgment, when consumers perceive that the quality, function and service of products have reached the expected goal, they will have higher perceived value. According to Chi and Yeh (2011), consumers can transfer their attitudes and feelings toward advertising spokespersons to products and create perceived value. Casal et al. (2008) pointed out that when consumers perceive the value of product availability, it is easy for them to positively evaluate the product, thus stimulating their potential demand. If the product can meet consumers' target demand, it will stimulate their purchase intention. In addition to consumers who have a demand for products, when consumers who have no demand for products perceive that products can bring them higher profits, they will also enhance their perception of the value of products, thus triggering a strong purchase intention (Zeithaml, 1988). To sum up, vloggers can promote consumers' understanding of product performance and improve their perception of value by selling and introducing products to the audience, and higher perceived value will contribute to higher purchase intention. Accordingly, this study puts forward the following hypotheses:

*H3a: Perceived value has a mediating effect between consumers' credibility perception of vloggers' two-sided messages persuasion and purchase intention*.
*H3b: Perceived value has a mediating effect between the appeal of vloggers' emotional persuasion and consumers' purchase intention*.
*H3c: The mediating effect of perceived value between the credibility of two-sided messages persuasion and purchase intention is greater than that between vloggers' emotional persuasion and consumers' purchase intention*.

## Research design

### Participants and procedure

Participants were recruited online with the help of the first author's colleagues and friends' assistance in forwarding the recruitment information of our study. Specifically, we made an online advertising poster that indicated the brief purpose and requirements of the study. Participants were required to have experience watching vloggers introducing products. Participants who read the recruitment information can scan the QR code, which would guide them to a page that specifically explains detailed content, and participation rules of the study. Participants were voluntary, and assured of full confidentiality. After giving the consent, participants could click the “next page” button and fill in the formal questionnaires. If they were unwilling to participate, they could close the page anytime. Each participant was rewarded 1.2 USD upon finishing the survey. The study was conducted in accordance with the principles of the Declaration of Helsinki and approved by the ethics committee of the first author's university.

Participants were asked to answer two screening questions about whether vloggers they watched had used two-sided messages persuasion (i.e., vloggers indicating both advantages and disadvantages of the products) or emotional persuasion (i.e., vloggers resorting to emotional arousals to promote the products). Only those who chose “yes” in both questions could continue filling in the following questionnaires that consisted appeal of emotional persuasion, credibility of two-sided messages persuasion, para-social interactions, perceived value of the products, purchase intention, and their demographic information. In an effort to minimize common-method bias, we used two different versions of the survey instruments by randomly determining the order of the measures (Ambrose and Schminke, 2009). Additionally, we added an attention check item (i.e., “please choose 2 for this item”) in the survey to guarantee the quality.

We received 532 returned responses. The initial screening revealed 7 repetitiveness and 5 incompleteness. We then removed 9 participants who answered the attention check items wrong, yielding 511 effective responses. One hundred and ninety participants (37.2%) were male, and 321 of them were female (68.2%). About 65.9% of the participants were between 21 and 30 years old, and 21.3% of them were between 31 and 40 years old. Regarding the platforms, about 62.2% of the participants indicated that they usually use Tiktok to watch vloggers' videos, 54.2% use Kuaishou, 64.2% use Weibo, and 63.1% use Xiaohongshu. Approximately 73% of them would watch food-related content, 58.3% of them cared about fashion, and 45.2% would watch electronic devices. About 7% of the participants never bought the products promoted by vloggers, 56.4% had 1 to 5 purchase experiences after they were persuaded by the vloggers they watched, 29.4% of them had 6 to 10 purchase experiences after vloggers' promotions, and 7.2% had more than 11 purchase experiences after being persuaded by vloggers. Additionally, 20.5% of them spent <100 Yuan for the most expensive single item advertised by vlogger, 48.1% of them paid 100–500 Yuan for the most expensive item, 24.9% of them spent 500–1000 Yuan for a single item, and 6.5% spent more than 1000 Yuan for the most expensive item they bought that was promoted by vloggers.

### Measure

A standard translation and back-translation procedure were followed (Brislin, 1976) to ensure that all the survey items were accurately translated from English to Chinese. All items measured in the survey were anchored to a 5-point Likert scale, ranging from 1 (strongly disagree) to 5 (strongly agree). It is worth mentioning that in order to ensure that participants can correctly understand the meaning of two-sided messages and emotional appeals, and make reasonable and realistic choices, we have made additional explanations next to the questionnaire items of two-sided messages and emotional appeals. For example: “When a vlogger promotes a product and informs the two-sided messages of the product (two-sided messages mean that both the advantages and disadvantages of the product are explained in the introduction), I would consider the vlogger a trustworthy person”; and “When a vlogger promotes a product and uses more emotionally stimulating (e.g., nostalgia: nostalgia for good things in the past; e.g., warmth: positive, emotional awakening about love, family and friendship; e.g., fear appeal: evoking people's sense of crisis and tension, like “after 30 years old, your body is not good, you need to supplement xx,” etc.), it will be more attractive to me. Thus, participants were selected on the basis of a complete understanding of all measurement items.

The credibility of two-sided messages persuasion was measured with adapted 4-item scale of Wang et al.'s (2017) Trustworthiness scale. An example was “The vloggers' two-sided messages persuasion made me feel that the advertisements had a trustworthy (dependable, honest, sincere, reliable) endorser.” Cronbach's alpha of the scale was.75 in the current study.

The appeal of emotional persuasion was measured with the adapted nine items based on the instrument introduced by Walters et al. (2012). An example item was “The vloggers' emotional persuasion made me fantasize about having the opportunity to use the product” Cronbach's alpha at was 0.94 in the current study.

Para-social interaction was measured with the 6-item Para-social interaction scale developed by Kim (2013). An example item was “I feel close enough to the vloggers to use his(her) social media platform.” Cronbach's alpha at was 0.77.

Perceived product value was measured with Sweeney and Soutar's (2001) 6-item Quality value scale. An example item was “This product has an acceptable standard of quality.” Cronbach's alpha was 0.84 in the current study.

Consumers' purchase intention was measured using Choi and Lee's (2019) 4-item Purchase Intention scale. An example item was “I would like to use the products that have been promoted by the vlogger on the video.” Cronbach's alpha at was 0.79.

Control variables. We controlled participants' age and gender in testing the hypotheses. Because prior research has shown that gender and age may be persuaded in different ways and thus develop different levels of purchase intention (Vilela and Nelson, 2016; Adaji et al., 2019).

## Results

### Analytical strategy

First, we conducted confirmatory factor analyses with Mplus 7.4 (Muthen et al., 2017) to examine whether the measurement scales represented distinct constructs. We then employed Path analyses in Mplus to test our hypotheses. Similar but more powerful than regression analyses, path analyses can simultaneously examine the parallel mediating effects (Muthen et al., 2017). Specifically, we first conducted Model 1 to test Hypothesis 1, and then in Model 2 we used bootstrapping to test and compare the parallel mediating effects of perceived product value and para-social interaction in the relationships of two types of persuasions and consumers' purchase intention. Finally, we conducted the supplementary analysis to test the common method bias issue, and tested an alternative model that excluded control variables to ensure the robustness of our model.

### Confirmatory factor analyses

As shown in Table 1, the hypothesized five-factor model exhibited a good fit to the data [χ^2^(333) = 898.76, *p* < 0.001, SRMR = 0.06, CFI = 0.92, TLI = 0.91, RMSEA = 0.06]. Furthermore, the five-factor model fitted better than other alternative models. Therefore, measures of the studied variables had good validity.

**Table 1:** Results of the confirmatory factor analyses (*N* = 511).

**Model**	***χ^2^ (df)***	**RMSEA**	**CFI**	**TLI**	**SRMR**	**Change from hypothesized model**
						***Δχ^2^ (df)***
1. Hypothesized 5-factor model	898.76 (333)	0.06	0.92	0.91	0.06	
2. Four-factor model (two independent combined together)	1237.90 (337)	0.07	0.87	0.85	0.08	339.14 (4)
3. Three-factor model (two independent variables combined together, and two mediators combined together)	1406.33 (340)	0.08	0.84	0.83	0.08	507.57 (7)
4. One-factor model (all five factors were combined into one factor)	1441.12 (342)	0.08	0.82	0.80	0.10	542.36 (9)

df, degrees of freedom; NNFI, Non-Normed Fit Index; CFI, Comparative Fit Index; RMSEA, Root Mean Square Error of Approximation.

### Descriptive statistics and correlations

Table 2 shows the means, standard deviations, and correlations among the studied variables. As expected, the credibility of two-sided messages persuasion is positively related to the consumer's purchase intention (*r* = 0.46, *p* < 0.001), and the appeal of emotional persuasion is positively related to the consumer's purchase intention too (*r* = 0.48, *p* < 0.001). Besides, both the credibility of two-sided messages persuasion and the appeal of emotional persuasion are positively correlated with perceived product value (*r* = 0.52, *p* < 0.001). Furthermore, the credibility of two-sided messages persuasion is positively correlated with para-social interactions (*r* = 0.48, *p* < 0.001), and the appeal of emotional persuasion and para-social interaction is positively correlated too (*r* = 0.57, *p* < 0.001). These correlations provide initial support for our hypotheses.

**Table 2:** Means, standard deviations, and correlations of the focal variables in study (*N* = 511).

**Variables**	***M***	***SD***	**1**	**2**	**3**	**4**	**5**	**6**	**7**
1. Age	2.29	0.51	-						
2. Gender	1.63	0.48	−0.13^**^	-					
Credibility of two-sided messages persuasion	3.84	0.65	0.13^*^	−0.02	(0.75)				
Appeal of emotional persuasion	3.44	0.95	0.24^**^	−0.13^**^	0.33^**^	(0.94)			
Perceived product value	3.81	0.73	0.13^**^	−0.06	0.52^**^	0.52^**^	(0.77)		
Para-social interaction	3.66	0.66	0.24^**^	−0.14^**^	0.48^**^	0.57^**^	0.59^**^	(0.84)	
Consumers' purchase intention	3.88	0.61	0.21^**^	−0.09	0.46^**^	0.48^**^	0.43^**^	0.65^**^	(0.79)

Age: 1 = over 20 years' old, 2 = 21–30 years' old; 3 = 31–40 years' old; 4 = over 40 years' old. Gender: 1 = male, 2 = female. Alpha internal consistency reliability coefficients appear on the main diagonal. Significance was determined using a two-tailed test.

* p < 0.05,

** p < 0.01.

### Hypotheses testing

Hypothesis 1 proposed positive relationships of credibility of two-sided messages persuasion (1a) and appeal of emotional persuasion (1b) with consumers' purchase intention. As shown in Model 1 of Table 3, the results showed that the credibility of two-sided messages persuasion was positively related to consumers' purchase intention (β = 0.33, *p* < 0.001), and the appeal of vloggers' emotional persuasion was positively related to consumers' purchase intention too (β = 0.34, *p* < 0.001). Therefore, Hypothesis 1a and 1b were supported. The direct effects model accounted for 33.0% variance of participants' purchase intention.

**Table 3:** Path analysis results of the direct and indirect effects (*N* = 511).

**Dependent variables**	**Model 1**		**Model 2**					
	**Consumers' purchase intention**		**Perceived product value**		**Para-social interaction**		**Consumers' purchase intention**	
**Measures**	***B (SE)***	***p***	***B (SE)***	***p***	***B (SE)***	***p***	***B (SE)***	***p***
Gender	−0.03	0.04	−0.01	0.03	−0.06	0.04	0.01	0.04
Age	0.08	0.04	−0.03	0.04	0.06	0.04	0.07^*^	0.03
Credibility of two-sided messages persuasion	0.33^**^	0.05	0.52^**^	0.05	0.23^**^	0.04		
Appeal of emotional persuasion	0.34^**^	0.06	0.33^**^	0.05	0.55^**^	0.05		
Perceived product value							0.26^**^	0.06
Para-social interaction							0.45^**^	0.05
*R^2^*	0.33^**^	0.04	0.48^**^	0.04	0.48^**^	0.05	0.40^**^	0.05
**Mediating effects**					**Indirect effect**		***LLCI***	***UCLI***
Credibility of two-sided messages persuasion → perceived product value → consumers' purchase intention					0.14^**^ (0.03)		0.07	0.20
Credibility of two-sided messages persuasion → para-social interaction → consumers' purchase intention					0.10^**^ (0.03)		0.05	0.15
Appeal of emotional persuasion → perceived product value → consumers' purchase intention					0.09^**^(0.02)		0.04	0.13
Appeal of emotional persuasion → para-social interaction → consumers' purchase intention					0.25^**^ (0.03)		0.18	0.32

Age: 1 = over 20 years' old, 2 = 21–30 years' old; 3 = 31–40 years' old; 4 = over 40 years' old. Gender: 1 = male, 2 = female. Significance was determined using a two-tailed test. Bootstrap sample size = 10,000. LLCI, lower level of the 95% confidence interval. UCLI, upper level of the 95% confidence interval.

* p < 0.05,

** p < 0.01.

Then we conducted Model 2 to test the mediating effects of para-social interaction and perceived product value and in the relationships of two kinds of persuasions and consumers' purchase intention. As shown in Table 3, the results showed that para-social interaction mediated the positive relationship between perceived credibility of two-sided messages persuasion with consumers' purchase intention (indirect effect = 0.10, 95% CI = [0.05, 0.15]), supporting Hypothesis 2a. Para-social interaction mediated the positive relationship between vloggers' appeal of emotional persuasion with consumers' purchase intention (indirect effect = 0.25, 95% CI = [0.18, 0.32]), thus Hypothesis 2b was supported. Additionally, the mediating effect of para-social interactions in the relationship between perceived credibility of emotional persuasion with purchase intention is more significant (difference of indirect effect = 0.07, 95% CI = [0.02, 0.12]) than that between perceived credibility of two-sided messages persuasion with purchase intention, supporting Hypothesis 2c.

**Figure 2:**
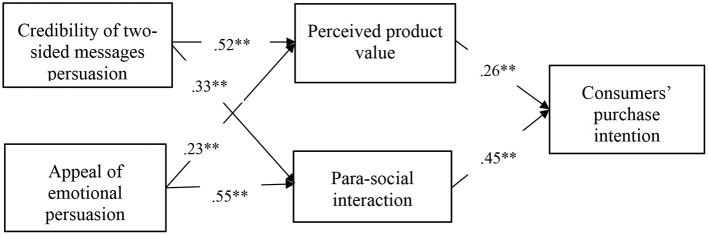
Standardized coefficients for the hypothesized model. ** = *p* < 0.001.

Simultaneously, results of Model 2 showed that perceived product value mediated the positive relationship between perceived credibility of vloggers' two-sided messages persuasion with consumers' purchase intention (indirect effect = 0.14, 95% CI = [0.07, 0.20]), supporting Hypothesis 3a. Besides, we found that perceived product value mediated the positive relationship between vloggers' appeal of emotional persuasion with consumers' purchase intention (indirect effect = 0.09, 95% CI = [0.04, 0.13]). Therefore, Hypothesis 3b was supported. Furthermore, comparing the mediating effects of perceived product value in the relationship between two different persuasions and consumers' purchase intention, we found that such mediating effect is stronger in the association of perceived credibility of two-sided messages persuasion with consumers' purchase intention than the appeal of emotional persuasion (difference of indirect effect = 0.06, 95% CI = [0.01, 0.12]). The parallel mediating model accounted for the variance of consumers' purchase intention. Therefore, Hypothesis 3c is supported. Figure 2 demonstrates the standardized coefficients of the integral model.

### Supplementary analyses

We addressed common method issue by creating a new construct of named common latent factor (e.g., Eichhorn, 2014). The result showed that the differences in standardized regression weights of constraint and unconstrained models were smaller than 0.20 for all the latent variables. Therefore, we assume that the current results were not substantially contaminated by the common method bias (e.g., Afthanorhan et al., 2021). Additionally, we re-ran the model without control variables of participants' age and gender, and the results remained unchanged. Specifically, we found the mediation of para-social interactions is more positive in the relationship between the appeal of emotional persuasion (vs. credibility of two-sided messages persuasion) with consumers' purchase intention (difference of indirect effect = 0.07, 95% CI = [0.02, 0.12]), and the mediation of credibility of two-sided messages is more positive in the relationship between two-sided messages persuasion (vs. appeal of emotional persuasion) with consumers' purchase intention (difference of indirect effect = 0.07, 95% CI = [0.02, 0.13]).

## Research discussion

### Conclusion

Starting from Aristotle's three appeals of persuasion, this study explores how vloggers' persuasion skills affect consumers' purchase intention, and draws the following conclusions:

First of all, vloggers' two-sided messages persuasion skills in promoting products have a significant positive impact on consumers' purchase intention. The results show that when vloggers use two-sided messages persuasion, it will increase the audience's trust in vloggers and their positive perception of products. This finding is consistent with previous studies. For example, Bohner et al. (2003) found in the study of advertising that two-sided advertising is more effective than one-sided advertising, because two-sided messages have two main functions when applied to advertising. First, it can increase consumers' perception of the credibility of information sources; second, it is the logical relationship between the relative importance of negative and positive information, which can promote consumers' favorable inference of essential attributes of products. This conclusion also confirms that two-sided messages can help explain Aristotle's persuasion theory of “ethos” and “logos.”

Secondly, the appeal of emotional persuasion of vloggers in promoting products significantly impacts consumers' purchase intention. The results of this study are consistent with previous research conclusions, which confirm the significant relationship between emotional persuasion and consumers' purchase intention. Some emotional appeals will affect consumers by changing the way the information conveyed in the appeal is processed (Achar et al., 2016). In this study, vloggers can stimulate and infect consumers by resorting to emotional expressions, and mobilize their different emotional perceptions of products, thus affecting their purchasing decisions.

Thirdly, para-social interaction mediates the relationship between vloggers' persuasive strategies and consumers' purchase intention, which is consistent with previous scholars' research (Jin and Ryu, 2020; Sokolova and Kefi, 2020). However, different from previous studies, this study draws a conclusion by comparing two-sided messages persuasion with emotional persuasion. When para-social interaction is used as an intermediary variable, compared with two-sided messages persuasion, vloggers' appeal to emotional appeal will make the audience feel stronger interaction with vloggers, produce common feelings and have stronger connection with each other, thus increasing the persuasion effect. That is to say, among Aristotle's three persuasion methods, the intermediary role of para-social interaction in “pathos” is greater than that of “ethos” and “logos.” Therefore, when vloggers cultivate a close and lasting connection, companionship, recognition, trust and attachment with the audience, it may be better to use emotional persuasion skills to promote products.

Finally, the mediating effect of perceived value in the relationship between vloggers' persuasive strategies and consumers' purchase intention has reached a significant level, indicating that vloggers' persuasive skills in promoting products will lead to consumers' perceived value, and then affect their purchase intention. This conclusion is consistent with the research of related scholars (Kwon et al., 2007; Gan and Wang, 2017). However, this study found that compared with emotional persuasion, perceived value plays a more obvious mediating role between vloggers' two-sided messages persuasion and consumers' purchase intention. In other words, when vloggers show credibility, professional knowledge and more abundant information of products by presenting the two-sided messages, they can make consumers feel that the value of products is higher. Therefore, the direct persuasion effect of two-sided messages is more excellent.

### Theoretical implications

First of all, this study combines classical Aristotle's theory with the contemporary social context, which can provide new ideas for understanding the social popular culture and phenomena. At the same time, this study also summarizes the connotation of Aristotle's persuasion theory and extracts variables convenient for measurement to construct an analytical model. This method expands the application scope of the original theory, and provides a reference for related research in the future.

Secondly, this study takes para-social interaction and perceived value as dual mediators, para-social interaction emphasizes emotional value, and perceived value tends to rational judgment. Therefore, this paper is to incorporate both emotional and rational traits of consumers into the study, which to a certain extent can help advertising parties better understand audience psychology.

### Practical implications

First of all, vloggers can better understand the audience's psychology, choose the proper marketing methods, establish a good and harmonious relationship with the audience, and enhance their influence. In the Internet era, various media have facilitated the life of the general public, and people have become more dependent on online information and tend to communicate through the media. At the same time, mass communication channels such as TV are replaced by online media channels in the media environment. Using the influence of key individuals or opinion leaders on media platforms to enhance consumers' brand awareness or purchase decisions has become a new marketing tool. Nowadays, the economic value of the Internet has attracted more and more people to become Internet celebrities, and all kinds of network information are jumbled, affecting the audience to make correct judgments. Therefore, it is particularly important for vloggers to adopt persuasion techniques to win the attention and trust of the audience and win the favor of brand owners. Based on para-social interaction and perceived value, this study explores how different persuasion strategies affect consumers' psychology, which helps vloggers adopt different persuasion methods for different types of audiences, and pay attention to the influence of different language styles on consumers' psychology. At the same time, attention is paid to using appropriate language rhetoric in advertisements, attracting potential customers, and guiding consumers to enhance their purchase intention.

Secondly, the characteristics of online vloggers determine that they can not only contact and influence a large number of consumers, but also reflect their marketing value through the creation and dissemination of content. Online vlogger marketing is a crucial paradigm for changing the traditional relationship between enterprises and consumers. This brand-new relationship not only changes the presentation mode of marketing information of commodity companies, but also derives a new mode of commodity value dissemination, which creates significant value for companies. Vloggers' different discourse persuasion skills enrich the forms of product display and marketing. For commodity companies or brand owners, this can not only improve sales performance, but also positively impact on consumers' brand attitude and purchase intention, which is conducive to enhancing product market share and brand image, and even consumers can further endorse brands based on personal social networks.

Finally, for consumers, they can quickly and accurately obtain product information based on vloggers' different language strategies, reduce the purchase risk, and at the same time satisfy their willingness to communicate with vloggers and meet their spiritual needs.

### Limitations

There are some limitations in this study, which need to be further discussed. First of all, there are many factors that language affects consumers' purchasing decisions, but this paper only discusses some of them, so the conclusion may not be comprehensive enough. Secondly, the current study used adapted scales to measure the credibility and appeal of two types of persuasion strategies, and future studies may first establish a solid scale or use experiments to test the hypotheses more rigidly. Thirdly, according to the data collected by the questionnaire, most of the participants are aged between 21 and 30, and the sample data are mainly young people, which is relatively limited. Future research can examine more people of different ages to improve and expand the sample data. Lastly, the model constructed in this study is a variable extracted from Aristotle's three appeals of persuasion theory, which may only partially summarize and explain Aristotle's rhetoric theory. Future research can build unique variables based on “ethos,” “logos” and “pathos” for measurement, and get more accurate results.

## Data availability statement

The original contributions presented in the study are included in the article/supplementary material, further inquiries can be directed to the corresponding author/s.

## Author contributions

XS and ZZ contributed to the hypothesis, collection of data, and writing of the first draft. WZ participated in the empirical work and analyzed the data. YH guided the direction of this study and put forward suggestions for the revision and improvement of the article. All authors contributed to the article and approved the submitted version.
